# Simulation of Runoff Changes Caused by Cropland to Forest Conversion in the Upper Yangtze River Region, SW China

**DOI:** 10.1371/journal.pone.0132395

**Published:** 2015-07-20

**Authors:** Pengtao Yu, Yanhui Wang, Neil Coles, Wei Xiong, Lihong Xu

**Affiliations:** 1 Research Institute of Forest Ecology, Environment and Protection, Chinese Academy of Forestry, Wan-Shou-Shan, Beijing, 100091, P. R. China; 2 Centre for Excellence in Ecohydrology, School for Environmental Systems Engineering, University of Western Australia, 35 Stirling Highway, Crawley WA 6009, Perth, Australia; Peking University, CHINA

## Abstract

The "Grain for Green Project" is a country-wide ecological program to converse marginal cropland to forest, which has been implemented in China since 2002. To quantify influence of this significant vegetation change, Guansihe Hydrological (GSH) Model, a validated physically-based distributed hydrological model, was applied to simulate runoff responses to land use change in the Guansihe watershed that is located in the upper reaches of the Yangtze River basin in Southwestern China with an area of only 21.1 km2. Runoff responses to two single rainfall events, 90 mm and 206 mm respectively, were simulated for 16 scenarios of cropland to forest conversion. The model simulations indicated that the total runoff generated after conversion to forest was strongly dependent on whether the land was initially used for dry croplands without standing water in fields or constructed (or walled) paddy fields. The simulated total runoff generated from the two rainfall events displayed limited variation for the conversion of dry croplands to forest, while it strongly decreased after paddy fields were converted to forest. The effect of paddy terraces on runoff generation was dependent on the rainfall characteristics and antecedent moisture (or saturation) conditions in the fields. The reduction in simulated runoff generated from intense rainfall events suggested that afforestation and terracing might be effective in managing runoff and had the potential to mitigate flooding in southwestern China.

## Introduction

Afforestation has been strongly encouraged in many countries such as China, South Korea, India, Nepal, and Bangladesh due to its multiple ecological benefits [1–4], for example reducing soil erosion and non-point source pollution, enhancing terrestrial and aquatic habitats, and increasing ecosystem carbon sequestration [5]. The annual planting rate was estimated to reach 4.5 million hectares globally [4]. In China, the largest plantation development country [4], six key forestry programs (SKFPs) has been implemented since 1998, targeting 76 million hectares of land for afforestation [6]. As a result, the total forest coverage in China has been increased from 13.9% of its land area in 1995 to 20.4% in 2009 and was expected to increase to 26% by 2050, as reported by the State Forestry Administration of China.

Afforestation, however, could lead to a tradeoff between water yield and biomass accumulation [7–9]. It often brought to substantial reductions in annual water yield, impacting downstream flows of the catchment [10–15]. The reduction in mean annual runoff after afforestation could be as high as 44% in humid regions [16], varying with tree species, age, rooting characteristics, and leaf area among watersheds [17–19]. It even varied within watershed depending on the site characteristics such as microclimate, soil, and landform [15]. The influence of these physical conditions on runoff may be so strong that the impacts of afforestation are masked [20]. Therefore forests are significant moderators in both the global hydrologic cycle and in the regulation of runoff at watershed scales [2]. As trees can access and transpire water from considerable depths in the soil, they are able to regulate surface runoff and groundwater recharge. Forest plantings in areas that were previously used for farmland generally generate less runoff because of high rates of growth and transpiration [13, 21, 22]. For example, the annual runoff reduction due to afforestation was estimated at between 50 to 300 mm/yr along an annual precipitation gradient from 400 to 3100 mm in China [23]. However, runoff did not always decline significantly when the trees or woody plants increased, for example in the Edwards Plateau region (Texas, USA), runoff did not decline and the contribution of baseflow doubled even though woody cover within the watershed expanded [24].

As one of SKFPs in China, the Grain for Green Project (GGP), also known as the “Conversion of Cropland to Forest Program”, is one of the world’s largest land-conservation programs [25]. In the past 10 years, some 9 million hectares of cropland has been converted to forests and 27 million hectares of forest plantations has been established through the GGP [26]. This kind of large-scale afforestation has many implications for the hydrology of watersheds and influences on extreme hydrological events such as droughts and floods [13, 22, 27]. In addition, land and water management practices, for example terraces constructed for sloping croplands, could also affect runoff and recharge in areas where afforestation was implemented through the GGP. Recently, it was reported that the GGP had reduced surface runoff and increase base-stream flow in the dry season in Northwest China, e.g. in the Loess Plateau and Inner Mongolian Plateau of China [28]. However, these studies mainly focused on the effects of vegetation conversion itself through comparing the water budget components on plots [28]. As a result, the influence of site condition changes associated with afforestation as part of the GGP (e.g. terracing) was not clear, making it difficult to fully evaluate the implications and impacts of the GGP.

The Guansihe River watershed in SW China, a first tributary of the Yangtze River, is a good example of evaluating the effect of GGP, as the area of cropland in the watershed was decreased by 11%, while forest area increased by 17% [29]. An ecological observation station, which includes 14 runoff plots and 8 permanent plots for vegetation studies, was established in this watershed in 1990 [30]. The hydrological effects of forests, such as soil physical characteristics [30], canopy interception [31], litter fall interception [32, 33], trunk stemflow [34], infiltration [35] and flow [36], were the subject of small scale (plot) case studies in this watershed since the implementation of the GGP. To assess the broader hydrological scale effects at the watershed scale, a physically-based distributed hydrological model, the Guansihe Hydrological Model (GSH model), was developed [37].

In this paper, we evaluate the hypothesis that the effect of afforestation on runoff response to rainfall is dependent on the site characteristics (paddy field or dry cropland), rainfall intensity and the placement of physical barriers on slopes (with or without terracing). The effects of land use change on watershed runoff were simulated by the GSH model based on 16 land use /land cover change scenarios for two rainfall events. These events were typical rainfall events that occur in this watershed, the first event, is defined as a high frequency medium intensity rainfall, the second event, a low frequency high intensity rainfall [37]. The aim of this case study was to quantitatively understand the hydrological effects of crop type and field terracing on runoff following conversion of degraded cropland to forest. This will assess the effectiveness of the GGP in mitigating the occurrence of flooding and provide data to develop an assessment framework for planned land use change at local and regional scales in mountainous areas of SW China.

## Data and Methods

### Study area

**Fig 1:**
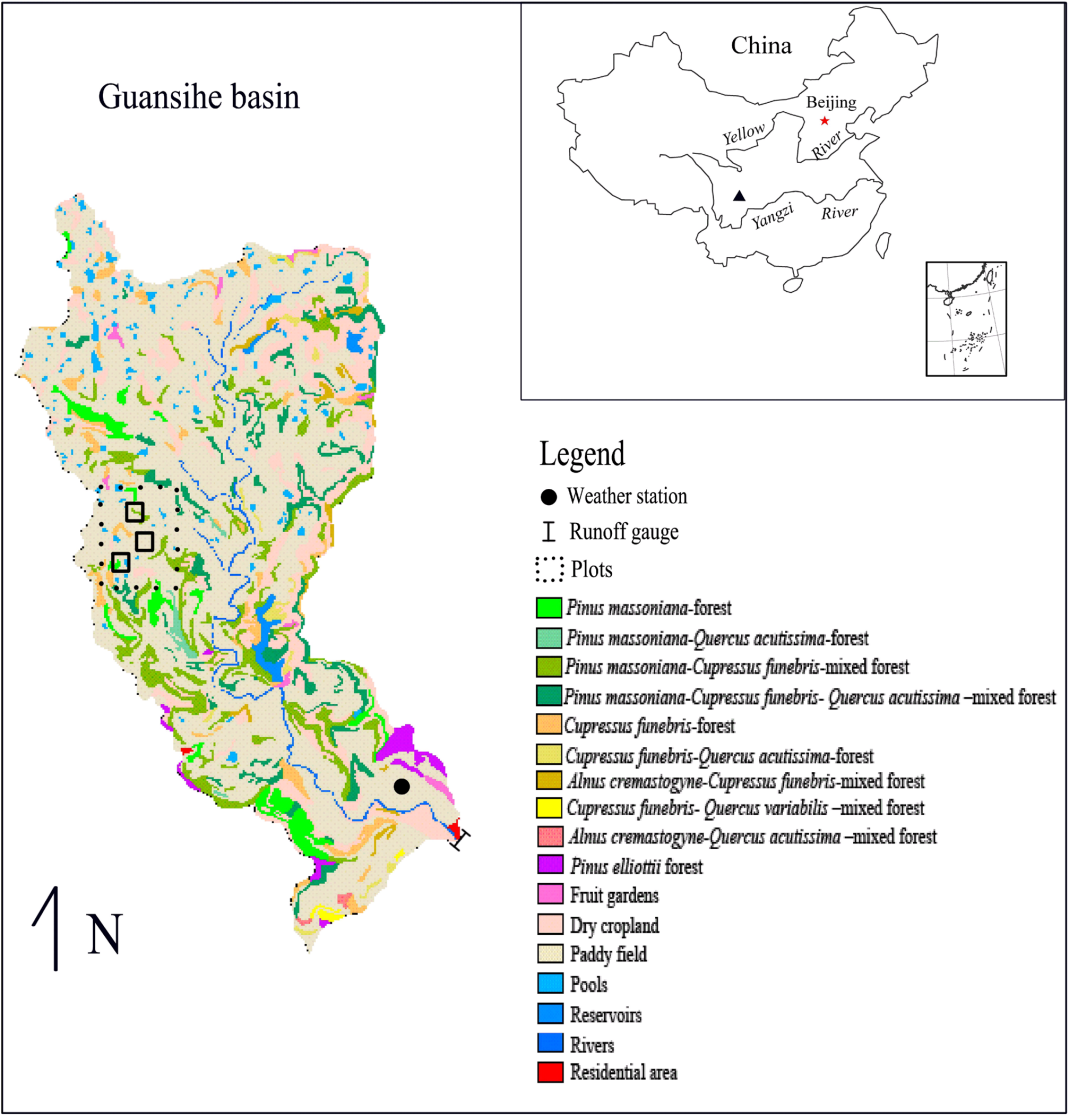
Location of the Guansihe watershed and its land cover.

**Fig 2:**
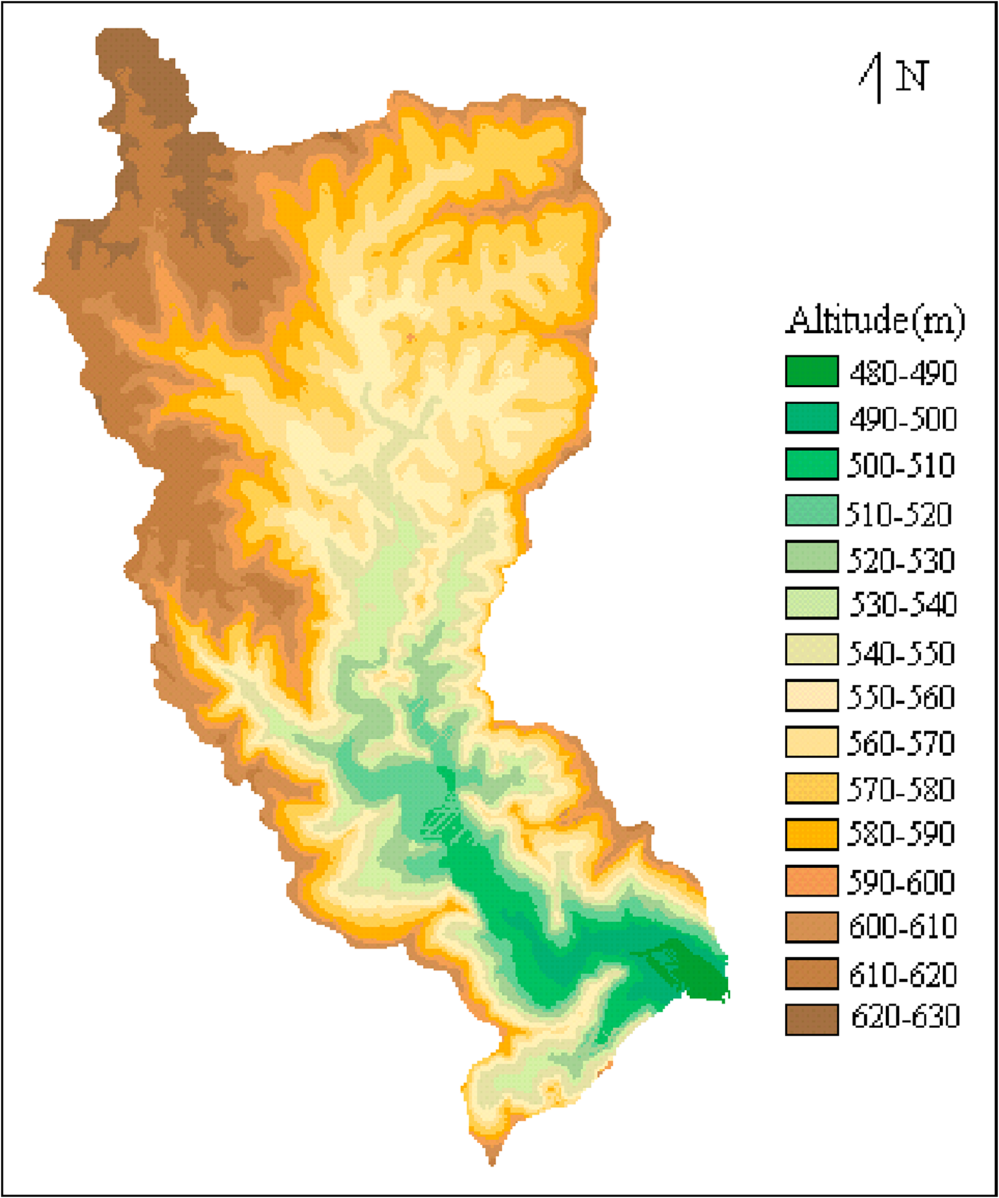
DEM of the Guansihe watershed.

All field observations were permitted to conduct at the 21.1 km^2^ Guansihe watershed (104° 46’─104° 49’ E and 31° 32’─31° 37’ N) by Youxian District Government of Mianyang City, Sichuan Province, China (Fig 1). The watershed lies in the upper reaches of the Yangtze River basin with landforms dominated by water-eroded hills. The elevations are between 480─630 m above sea level (a.s.l.) (Fig 2). Haplic acrisol, chromic cambisol and eutric regosol are the typical soils of this region of SW China and are the major soil types within the Guansihe watershed. The climate is classified as northern subtropical humid monsoonal, with a mean annual temperature of 16.1°C, mean annual relative humidity of 79% and mean annual precipitation of 921 mm occurring mainly in summer and autumn [34].

The agriculture and forest ecosystems of this watershed are representative of the region. The existing forests cover 16.7% of the watershed and are comprised of secondary forests and artificial plantations that are mainly consisted of the following species: *Cupressus funebris*, *Pinus massoniana*, *Alnus cremastogyne*, *Quercus acutissima* and *Quercus variabilis* (Fig 1). The community structure is simple, with prominent layers of trees, shrubs and herbs. The most common shrub species found in the watershed are *Vitex negundo*, *Myrsine africana*, *Pyracantha fortuneana* and *Elaeagnus pungens*. The herbs species are dominated by *Eleusine indica* and *Imperata cylindrica* var. *major*.

**Fig 3:**
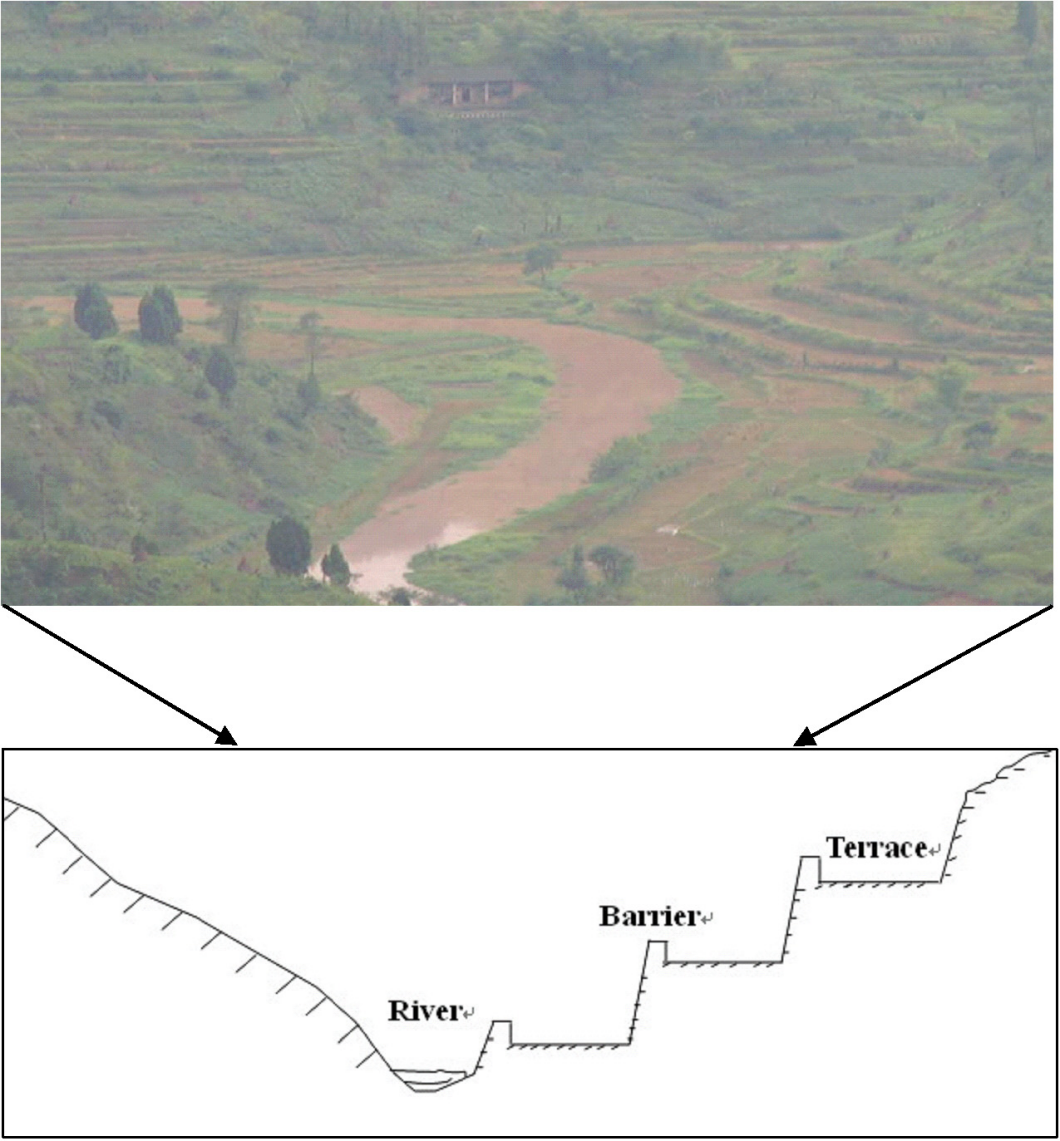
The distribution of terraced fields and its profile.

In this watershed, all the croplands, which accounts for 72.2% of the watershed area, were terraced. As small pieces of flat land, these terraced fields were constructed along contour lines on the hill slopes and enclosed by about 20-cm-high walls (Fig 3). In this watershed, 81.7% of the terraced fields are used as paddy fields that are flooded, with only the terraced fields on the upper slopes being used as dry cropping systems for growing maize, beans and other crops.

### Structure of the GSH model

As a physically-based distributed hydrological model, the GSH model was developed to account for the spatial heterogeneity of physio-geographical elements in this watershed through simulating the hydrological processes that occur before, during and after a rainfall event [37]. For each simulation, the watershed was divided into homogeneous cells with unique environmental settings. The size of the cell indicates the spatial resolution, i.e. a smaller cell has higher resolution and describes the processes or watershed functions in more detail.

Each cell has a system of vegetation and soil, and has layered hydrological functionality that includes interception, evaporation, transpiration, and infiltration. The vegetation complexes were divided into four layers (trees, shrubs, herbs, and litter) and the soils divided into two layers (root zone and ground layer).

**Fig 4:**
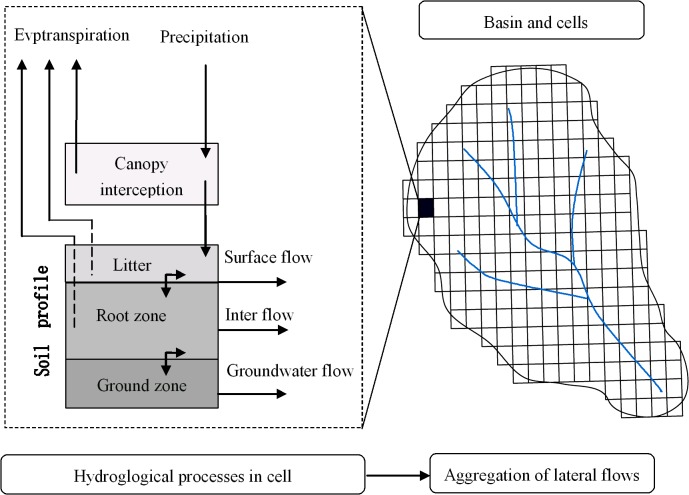
The systemic frame of the GSH model.

Hydrological processes that are the water movements through different layers of the same cell as well as transfers between the neighboring cells within the same layer were tracked continuously by the GSH model. Its systemic frame was shown in Fig 4. The GSH model calculates interception, transpiration and evaporation using the Shuttleworth–Wallace equation, an improved Penman–Monteith approach.

The soil-water characteristics are defined using the approach by Brooks and Corey [38]. Surface runoff is calculated using Philip’s infiltration equation [39, 40], kinematic wave equation and Manning’s formula [41]. The water movement through the soil is simulated with the Darcy-Richards equation [38].

The GSH model runs at a minute time-steps. Its input is rainfall and output is runoff [37].

### Data preparation

Topographic maps of Guansihe watershed with a scale of 1:10000 were selected as an appropriate scale to capture physio-geographical characteristics. The maps were scanned and digitized to construct a digital elevation model (DEM) (Fig 2). Each grid in the DEM was defined as a cell in this study. The Guansihe watershed was divided into 7,378 cells of size 50 m × 50 m. The vertical resolution was 0.25 m.

A land use map (scale 1: 10,000) and a forest map (scale 1:10,000) were scanned, digitized and transformed from vector form to raster form at 50 m × 50 m resolutions. The maps were georeferenced with the DEM. Spatial vegetation data layer was applied to this data. The vegetation type of each cell and the forest management sub-compartments was then derived. Furthermore, the characteristics of cells, e.g. biomass, leaf area index (LAI), soil type and its depth was attributed to each cell using available forest survey data obtained by local forestry department. The parameters, such as interception storage capacity, water-holding capacity of litter, and soil porosity (Table 1) were assigned to each cell based on vegetation type and data obtained from instrumented plot trials [30].

**Table 1:** The soil characteristics under different vegetation types.

Vegetation types	Bulk density (g cm^-3^)	Field capacity (%)	Capillary capacity (%)	Saturated water content (%)
*Pinus massoniana*-forest	1.46	22.3	25.8	27
*Cupressus funebris*-forest	1.24	29.5	34.3	39.6
*Cupressus funebris-Quercus acutissima*-forest	1.46	21.2	24.7	28.4
*Pinus massoniana-Quercus acutissima*-forest	1.5	20.3	24.2	25.5
*Alnus cremastogyne-Cupressus funebris*-mixed forest	1.29	29.2	33.8	37.5
*Alnus cremastogyne-Quercus acutissima–*mixed forest	1.46	21.2	24.7	28.4
*Pinus massoniana- Quercus acutissima–*mixed forest	1.47	20	23.5	24.3
*Pinus massoniana- Cupressus funebris- Quercus acutissima*–mixed forest	1.47	20	23.5	24.3
*Cupressus funebris- Quercus variabilis–*mixed forest	1.46	21.2	24.7	28.4
*Pinus elliottii-*forest	1.43	18	23.5	24.8
Dry croplands	1.39	24.4	29.4	31.2
Paddy fields	1.39	24.4	29.4	31.2

### Model calibration and validation

At Mianyang meteorological station (about 15 km downstream the Guansihe watershed), the rain days with precipitation > 10 mm was recorded and averaged as 217 days per year between 1970─2010. A maximum daily precipitation of 215 mm was recorded during this period. Surface flow is usually generated when the rainfall is greater than 10 mm at plot scale [36]. Thus, in order to calibrate and validate the GSH model, the rainfall-runoff response to four rainfall events with precipitation totals ranging from 16.9 mm to 206 mm with variable rainfall intensities were used (Table 2 & Fig 4). The recorded runoff data and the GSH model simulations were then compared.

**Table 2:** Details of the four rainfall events selected for testing of the GSH Model.

	Precipitation (mm)	Duration (h)	Max. rainfall intensity in five minutes (mm/hr)	Average Recurrence Interval (ARI) of the precipitation in 24 hr (a)	Precipitation of last rainfall event (mm)
Extreme rainfall	206	42.5	115.8	30	24.1
Medium rainfall	90	35.6	40.2	1	65.0
Small rainfall	20.3	37.9	30.0	1	26.2
Small rainfall	16.9	32.2	3.0	1	1.6

The absolute error (A) and relative error (B) were used to validate the model simulations using (Eq (1) and (2)),

$$A=\left(\sum_{i=1}^{n}\left|MW_{i}-SW_{i}\right|\right)/n$$ (1)

$$B=\left(\sum_{i=1}^{n}\left(\left|MW_{i}-SW_{i}\right|/MW_{i}\right)\right)/n$$ (2)

where *MW*_*i*_ is measured runoff (m^3^/s) at the outlet of the watershed, *SW*_*i*_ is the runoff (m^3^/s) calculated by the GSH model, and n is the number of measurements.

The non-dimensional efficiency criterion of Nash and Sutcliffe (*E*) (Eq (3)) [40] were also used to evaluate the quality of model simulation

$$E=1-\frac{\sum {\left(MW_{i}-SW_{i}\right)}^{2}}{\sum {\left(MW_{i}-MW_{av}\right)}^{2}}$$ (3)

where the variable *MW*_*av*_ describes the mean observation value during the simulation period. *E* can vary from minus infinity to 1, the latter corresponding to a perfect fit.

### Scenario design

In mountainous regions such as the Guansihe watershed, croplands are commonly located on relatively steep slopes due to the limited availability of flat land. Areas with steeper slopes are more likely to be chosen for afforestation, since steeper slopes are more prone to soil erosion. In this case study, four afforestation scenarios using mixed forest *Pinus massoniana-Cupressus funebris* plantations are defined, based on the slope angle. Scenarios I-IV are categorized as land with slope angles of >15°, 10─15°, 5─10° and <5°, respectively (Table 3). A further four options are defined within these scenarios to identify changes in runoff from terraced or un-terraced slopes with different cropping systems, for example rice paddy or dry (rainfed) cropland (Table 3). These options create a four by four matrix providing a set of 16 scenarios associated with cropping systems, terraces, afforestation and slope categories. In these scenarios, all croplands in the corresponding cropping systems in each slope angle class were converted to forests.

**Table 3:** Description of model scenarios.

Scenario set No.	Slope	Scenario No.	The type of cropland converted to forest	Total area of Cropland converted to forest *	Terrace retained?
I	>15°	1	Dry cropland	4.5%	Yes
		2	Paddy field	8.9%	Yes
		3	Dry cropland	4.5%	No
		4	Paddy field	8.9%	No
II	10-15°	5	Dry cropland	4.1%	Yes
		6	Paddy field	16.1%	Yes
		7	Dry cropland	4.1%	No
		8	Paddy field	16.1%	No
III	5-10°	9	Dry cropland	2.8%	Yes
		10	Paddy field	18.6%	Yes
		11	Dry cropland	2.8%	No
		12	Paddy field	18.6%	No
IV	<5°	13	Dry cropland	1.9%	Yes
		14	Paddy field	15.3%	Yes
		15	Dry cropland	1.9%	No
		16	Paddy field	15.3%	No

*Total area means the percentage of the corresponding type in the whole watershed area.

For each scenario simulation, the cells converted from cropland to *Pinus massoniana-Cupressus funebris* mixed forest; the vegetation parameters are modified to reflect the changed vegetation cover, but the soil parameters remained unchanged, given the fact that soil physical characters change slowly after afforestation [30].

Two rainfall events observed by weather station (Fig 1) in Guansihe watershed, which were with precipitation totals of 90 mm (medium rainfall) and 206 mm (extreme rainfall) were chosen to simulate the effect of afforestation on runoff response (Table 2).

### Assessing the hydrological effects of changed land use

Afforestation only occurred in part of the watershed, rather than over the whole one [29]. In this study, the simulated rainfall-runoff responses generated from areas based on 16 land use change scenarios are analyzed. The simulated values of watershed runoff were compared with the runoff records for events in the Guansihe watershed with the existing vegetation. The simulated responses were then compared for the same rainfall events with 10% of watershed area converted to forest using 16 scenarios and the effect on response was assessed.

## Results

### Model calibration and validation

**Fig 5:**
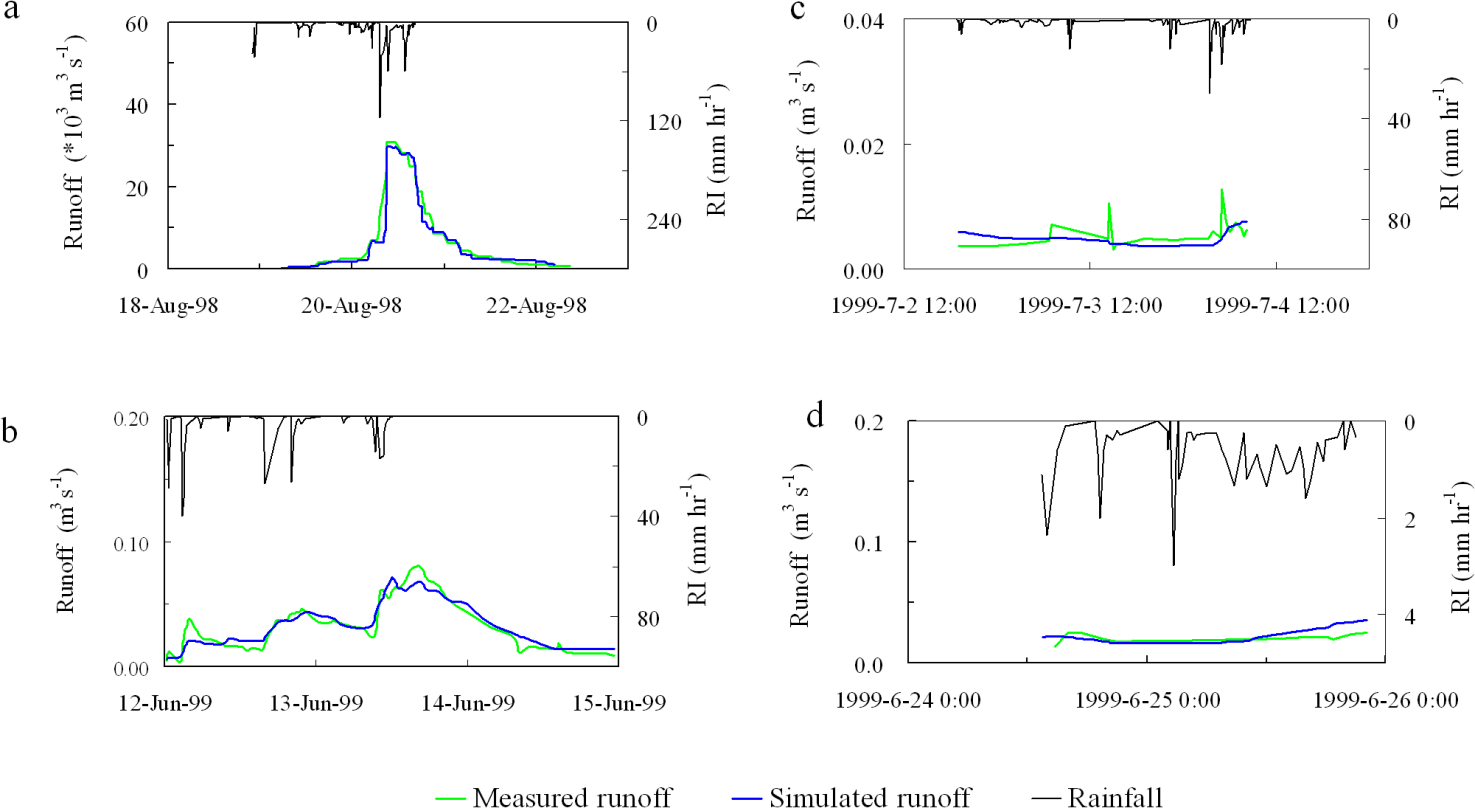
The comparison of hydrographs measured and simulated by the GSH model under four simulated rainfall events. The total precipitation of rainfall event in a, b, c, and d were 206 mm, 90 mm, 20.3 mm and 16.9 mm, respectively; RI is rain intensity.

The hydrograph simulated by the GSH model was compared to the observed data as shown in Fig (5A–5D). Under low intensity rainfall (i.e., 16.9 mm and 20.3 mm rainfall events), no peak in discharged is observed for either the measured or GSH-simulated hydrographs (Fig 5D). Small discharge peaks observed in Fig 1C were not simulated by the GSH model owing to the very low discharges (<0.02 m^3^s^-1^). These could be viewed as the base flow of the original hydrograph. Note that the relative errors of the simulated total runoff are less than 10% (Table 4). Under more intense rainfall (90 mm, with an average recurrence interval (ARI) of 1 year), the relative errors in simulated peak discharge and total runoff are 8.5% and 0.9%, respectively. Under extreme rainfall (206 mm, with an ARI of 30 years), the simulated hydrograph displays a relative error of 3.8% in peak discharge and 13.6% in total runoff (Table 4). The non-dimensional efficiency criterion of Nash and Sutcliffe (E) was 0.88 for rainfall of 90 mm and 0.92 for rainfall of 206 mm. These results demonstrate that the calibration and validation of the GSH model was representative of watershed response.

**Table 4:** Comparison between measured runoff and that simulated by the GSH Model.

Errors in simulated runoff per rainfall event	Calibration	Validation		
	Rainfall of 20.3 mm	Rainfall of 16.9 mm	Rainfall of 90 mm	Rainfall of 206 mm
Relative error of peak discharge (%)	No clear peak	No clear peak	+8.5	+3.8
Relative error of total runoff (%)	-1.1	+8.0	+0.9	+13.6

### Effect of cropland afforestation on runoff

**Fig 6:**
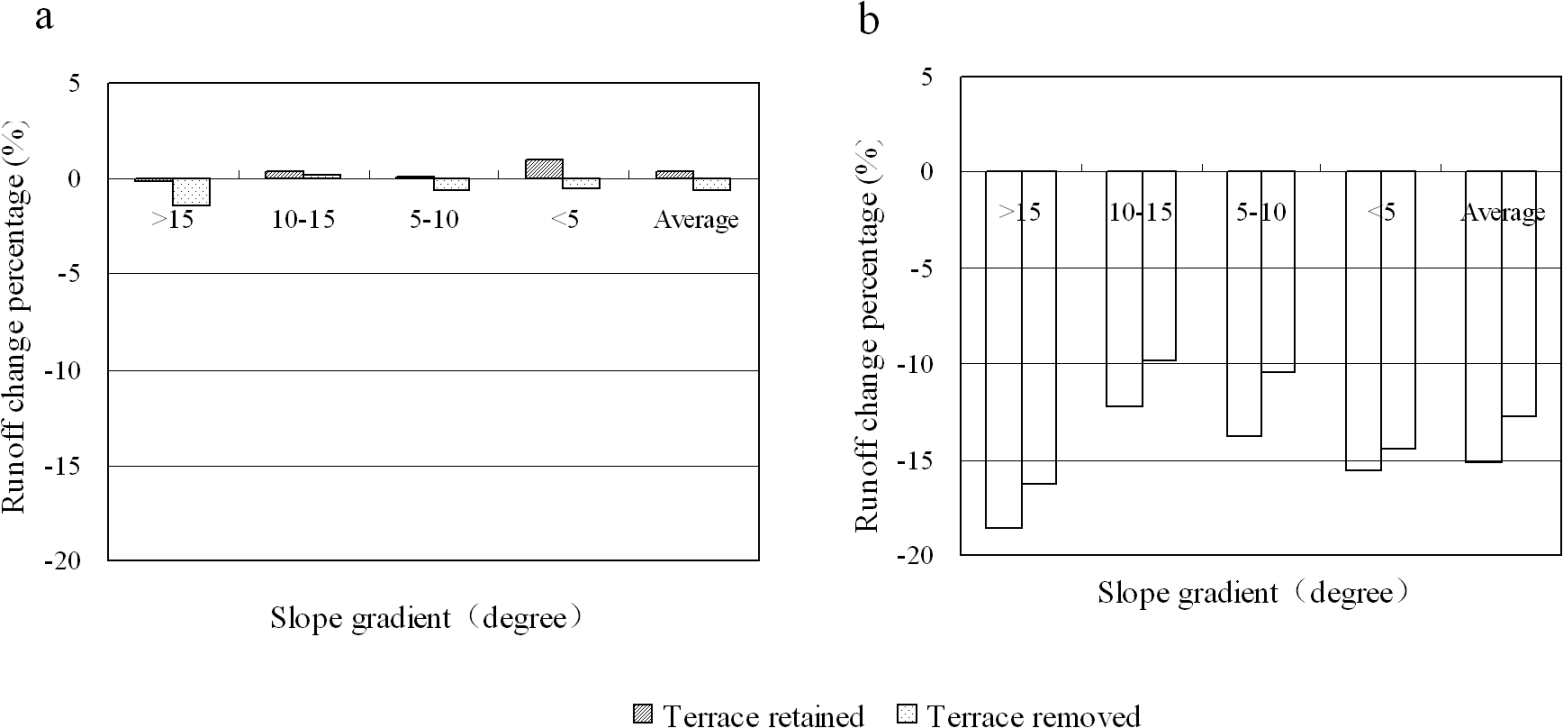
Runoff change for the 90 mm rainfall event when cropland in 10% of watershed area was forested. The runoff changes were expressed as the percentage of watershed runoff under the current land cover (a: dry cropland converted to forest; b: paddy field converted to forest).

The conversion of dry cropland to forest did not greatly impact on watershed runoff generation. For example, when dry cropland is converted to *Pinus massoniana-Cupressus funebris* mixed forest, the variation in runoff observed for the simulations ranged from -0.2% to +1.0% (Fig 6). The variability illustrated in this simulation is close to the relative error inherent in the GSH model (see Table 4). For the simulation using the 206 mm rainfall event, runoff response decreased by 1.0% when cropland on slopes with slope angle greater than 15° was converted to *Pinus massoniana-Cupressus funebris* mixed forest (Table 5).

**Table 5:** Percentage change in runoff and peak flow for the 206 mm rainfall event when cropland in 10% of watershed area with slope angle > 15° was forested. The runoff changes were expressed as the percentage of watershed runoff under the current land cover. Additionally, all the values were scaled as the runoff change when the cropland in 10% of watershed area was forested. "+" and "-" show increase and decrease, respectively.

Hydrographical parameters	Dry cropland converted to forest		Paddy field converted to forest	
	Terrace retained	Terrace removed	Terrace retained	Terrace removed
Runoff	-1.0	5.4	-7.2	-6.4
Peak flow	-1.0	4.4	-11.4	-9.6

By contrast, the afforestation of paddy fields was found to greatly reduce watershed runoff under medium (90 mm) rainfall event, with reductions of between 9.8% and 18.6% depending on the slope angle (Fig 6). The greatest reduction occurred on slopes with slope angle greater than 15^o^. The watershed runoff under the extreme rainfall event (206 mm) decreased by 7.2% when paddy fields on slopes with slope angle greater than 15° were converted to *Pinus massoniana-Cupressus funebris* mixed forest, with peak flow declining by 11.4% (Table 5). These indicated that the conversion from paddy fields to forests would cause a great reduction of watershed runoff.

### Effect of terraced fields on runoff

The influence of terraced fields on runoff generation varied with the rainfall intensity. For the medium rainfall event, the variation in total runoff was less than 1.5% after dry cropland to forest conversion (Fig 6). However, for the extreme rainfall event, both the total runoff and peak flow declined strongly for the terraced relative to un-terraced scenarios (Table 5). For example, when dry cropland on slope angles more than 15° were converted to *Pinus massoniana-Cupressus funebris* mixed forest, the peak flow in the scenario with terraces was 6.4% lower than that without terraces, while total runoff displayed a 5.4% decline (Table 5). After paddy field to forest conversion, the runoff from terraced land was 2.3% less than land without terraces for the 90 mm rainfall event (Fig 6). The runoff from terraced land was 0.8% less than that from un-terraced land, and the peak flow with terraces was 1.8% lower than that without terraces for the simulation of runoff generated by the 206 mm rainfall event (Table 5).

The inclusion of terraces in the simulation resulted in an average 15.1% decrease in total runoff generated from the 90 mm rainfall event (Fig 6) and an 11.4% decrease for the 206 mm event (Table 5). This decline in total runoff was observed in the simulation of the conversion of paddy fields to forest within the watershed. Although the total runoff reduction in percentage for the 206 mm rainfall event was less than the 90 mm event, its decrease in volume was 2.2 times greater than the 90 mm event. This represents a significant reduction in the total runoff generated within the watershed during an extreme event, and is likely to impact on the severity and extent of flooding within the lower valley.

## Discussion

Our study showed that the effect of cropland to forest conversion on watershed runoff was very sensitive to original land use, i.e. paddy field or dry cropland. The big difference between dry cropland and paddy fields was their soil moisture. In paddy fields, the soil is always saturated and there may even be stored with surface water during the growing season, which coincides with the rainy season. On the contrary, in dry cropland, soil moisture is commonly lower than the field capacity and there is normally no surface water. Forest effect on runoff depends mostly on soil depth, structure and degree of previous saturation of soil before rainfall events [2, 42]. In our study area, runoff yield was determined by the level of antecedent soil moisture and soil storage capacity, which is not generated until the soil is saturated by rainfall, particularly for the dry cropland areas [43]. This effect of antecedent soil moisture on runoff was also observed in the field at both the plot and small watershed scale [44]. Accordingly, we conclude that this substantial decrease in runoff after cropland to forest conversion is likely due to the significant soil moisture differences experienced between paddy fields and forested environments. Conversion of the dry cropland to forest would not result in a significant change in the soil saturation capacity, but would increase vegetative storage capacity (of ~5 mm) of interception [33]. Consequently, the watershed runoff after vegetation conversion from dry cropland to forest is largely unchanged for the simulated rainfall events (Fig 6 &Table 3).

Therefore, the influence of afforestation should be evaluated according to the variation from the original land use type (e.g. dry cropland or paddy field). When dry cropland is forested, its effect on flooding is very limited, whereas when paddy fields are forested, the watershed runoff volumes and peak flows are strongly reduced. Paddy fields are the most common land use type in the study area, accounting for 81.7% of cropped area in the Guansihe watershed. Our simulation results suggested that these areas should been preferentially designated as the priority land use change for GGP in mountainous regions such as those in SW China. However, these paddy fields in South China supply more food for China's huge population and it was worried that the widespread conversion from croplands would threaten future food security of China. Further studies are required on where and how much of paddy fields should be afforested.

Factors other than land uses, such as terrace and slope angle, can also mitigate changes in runoff magnitudes following cropland to forest conversion. For example, the presence of terraces in dry cropland did not change the runoff response for the 90 mm rainfall event. The contrasting terrace effect between dry cropland and paddy field indicated that terraces did not affect runoff until the localized soil and terrace water storages were at capacity (or saturated) at the ground surface, such as in the paddy fields. Compared with the effect of terracing on the Loess Plateau where is the most popular area to control soil and water erosions in China [45], the terrace effect in our study region had a lower impact. In the Loess Plateau, runoff reduction from terraced cropland was measured from 46.9% in dry year to 100% in wet year [46, 47]. The influence of terraces on runoff from paddy fields accounted for approximately 20% of the impact of afforestation in our study, which was suggested to be related to the processes generating runoff, and the rainfall characteristics. In the Loess Plateau where is located mainly in the semiarid region, the runoff is generated due to infiltration-excess runoff [48]. However, this runoff generated from the Guansihe slopes occur when a thin layer of saturation occurs at the surface rather than saturation of the whole soil profile [49]. In the Loess Plateau, the rainfall is generally captured earlier and stored until the terrace’s water holding capacity is reached and runoff is generated. In these conditions the runoff was generated from areas that were not saturated but occurs when infiltration and storage capacity had been exceeded due to the rainfall intensity and duration experienced during large events. The terrace effect should therefore be evaluated according to regional factors such as climate rather than just those characteristics of the terracing itself. The slope angle was used as key factor of surface runoff and soil erosion control by most related studies [50]. It also has been employed in forestry plans as a key factor in determining areas to undertake forestation, e.g., in the GGP [6]. It was found that surface flow will increase following slope angle increasing on plot scale [50]. But in our study, the watershed runoff did not keep increasing when steeper slopes were forested. It appeared a complex of effects of several factors such as original vegetation, terracing, and rainfall existed.

Our study implied that the conversion of cropland to forest can potentially contribute to flood management. The suggested link between afforestation and flooding is thought to be weak and has been highlighted in some reports [8, 21]. For example, it was summarized by the Food and Agriculture Organization of the United Nations (FAO) that forests have only a limited influence on major downstream flooding, especially for large-scale events [42]. On a local scale, forests and forest soils are capable of reducing runoff only for small-scale rainfall events, which are not responsible for severe flooding in downstream areas [42]. Although there was no direct evidence on the effects of the GGP on flooding in China, the GGP was suggested to strongly reduce soil erosion, for example, soil erosion was reduced by 320 million tons in sum from 1999 to 2009 in Sichuan Province, China [29]. Decreases in soil erosion would also likely benefit flood mitigation through increasing the effective capacity of the river channel. This effect will probably be particularly enhanced in regions where steep slopes exist, such as the Guansihe watershed. Therefore, the quantitative estimations should be partitioned to determine the explicit contributions to flood alleviation from the direct effects, for example interception reduction and transpiration increase, of cropland to forest conversion, and the effects from decreased soil erosion.

## Conclusions

Our study tested the hypothesis that the level of influence of afforestation on runoff depends on site factors, for example antecedent soil conditions, land use, and physical barriers on slopes (e.g., terraces). Analysis of the hydrological and land-use data from the Guansihe watershed indicate that this influence on runoff differs strongly between land use types such as dry cropland and paddy fields. The influence of paddy field to forest conversion was stronger than that on dry cropland, due to more soil moisture in paddy fields. The terracing of cropland could enhance this effect on runoff reduction. But these effects reduced under extreme strong rainfall. The simulated runoff reduction implied that the conversion of paddy fields to forests could potentially mitigate flooding.
